# Benign Metastasizing Leiomyoma: New insights into a rare disease with an obscure etiopathogenesis

**DOI:** 10.1186/s13000-023-01427-4

**Published:** 2024-01-03

**Authors:** Aziz Chouchane, Sassi Boughizane, Monia Nouira, Sami Remadi

**Affiliations:** 1 Institute of Tissue Medicine and Pathology, University of Bern, Bern, Switzerland; 2 Service de Gynécologie, Hôpital Farhat Hached, Sousse, Tunisia; 3 Clinique Les Oliviers, Sousse, Tunisia; 4 Laboratoire d’Anatomie Et Cytologie Pathologiques, Rue Leopold Senghor, 4000 Sousse, Tunisia

**Keywords:** Benign Metastasizing Leiomyoma, Ethiopathogenesis, Coelomic tissue, Metaplasia

## Abstract

**Background:**

Benign metastasizing leiomyoma (BML) is a rare disease with an unknown etiopathogenesis that mostly affects middle-aged women with uterine leiomyoma. Many metastatic nodules outside the uterus characterize the condition. The metastases are smooth muscle lesions without malignancy. Morphologically and immunohistochemically, they resemble uterine leiomyomas, indicating a shared clonal origin. The lungs are the most prevalent site for incidental metastasis detection. BML has a relatively slow progression and good prognosis, and historically, there has been a lack of established guidelines for its treatment.

**Case presentation:**

Herein, we report a case of BML in a patient with multiple metastases. Through extensive histological and immunohistochemical analyses, this complex case enabled not only the definitive diagnosis of BML, but also shed light on its complex etiopathogenesis.

**Conclusion:**

This study presents novel histology evidence suggesting a potential causal relationship between metaplasia and the development of BML.

## Background

Human leiomyomata (fibroids) are the most common and benign uterine smooth muscle tumors in reproductive-age women [1–3]. Benign uterine leiomyomas rarely develop intravascular, disseminated peritoneal, or metastatic patterns [4].

Benign metastasizing leiomyoma (BML) is a rare and poorly understood clinicopathological condition that impacts women who have a previous medical history of uterine leiomyomas. The disease is distinguished by the appearance of several nodules that correspond to histologically benign metastatic deposits of smooth muscle tissue in various sites outside the uterus [5–9].

Here, we present a unique and complex case of BML diagnosed in a patient who exhibited the presence of many metastases. Notably, we present, for the first time, the key histological findings that provide insight into the etiology of this disease, hitherto ill-known.

## Case report

A 43-year-old woman, Gravida 3, Para 3, complained of severe lower abdominal pain, mostly in the left hypochondrium, for six months. The patient's medical and gynecological history was unremarkable, except a myomectomy for a benign leiomyoma performed 12 years ago. The physical examination revealed a bloated abdomen, a palpable left hypochondrium mass, and an enlarged uterus. These findings suggested leiomyoma or adenomyosis.

**Fig. 1:**
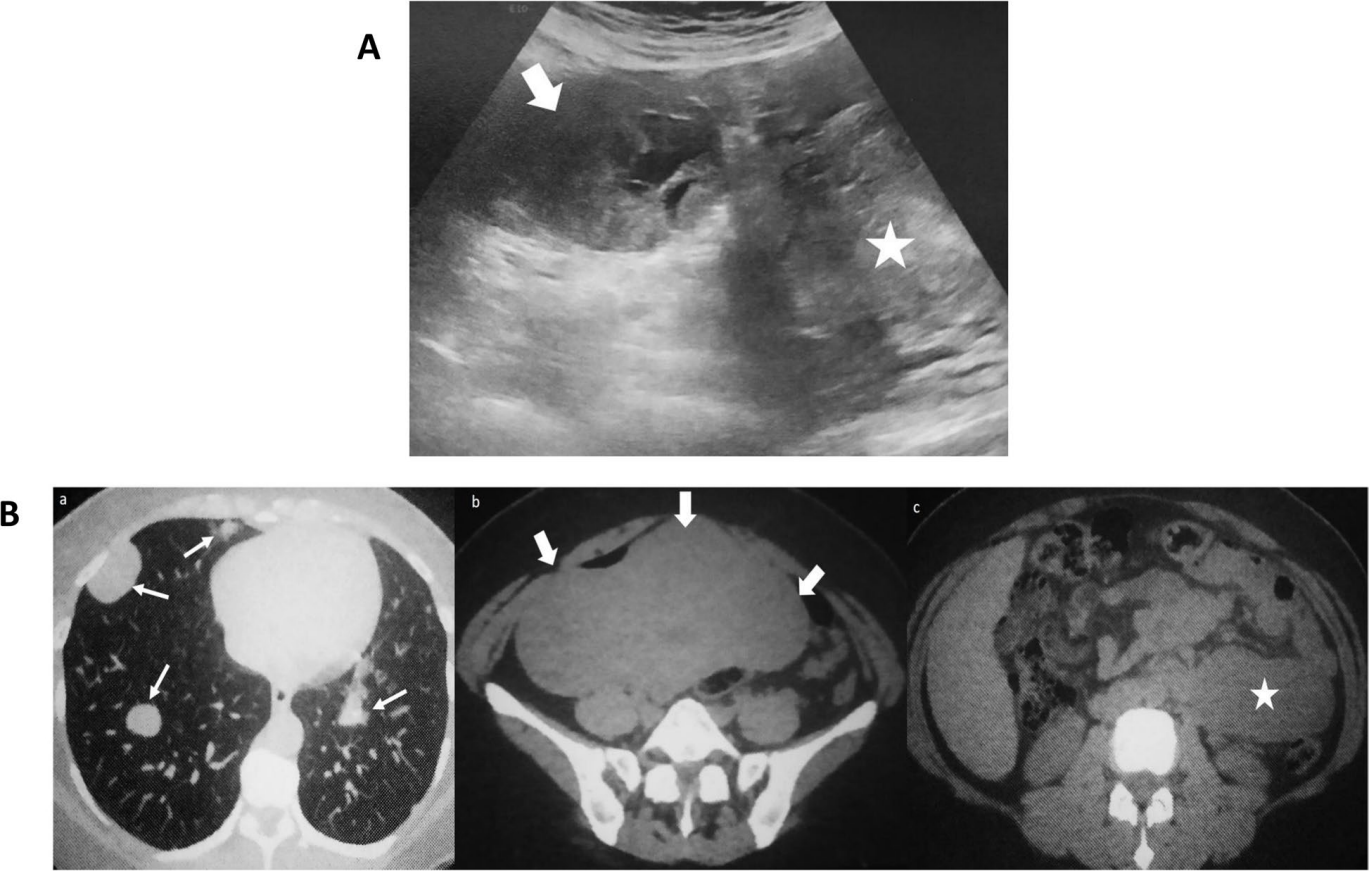
**A** An ultrasound image of a heterogeneous hypoechoic subserosal nodule (arrow) in the uterus (indicated by ★) is shown.** B** A non-enhanced CT scan demonstrating (**a**) many well-defined lung nodules (arrows), (**b**) multiple uterine fibroids (thick arrows), and (**c**) a mass in the left hypochondrium (indicated by ★)

The abdomen ultrasound revealed multiple large and heterogenous uterine nodules, the largest measuring 7 cm. Size and appearance were normal for both ovaries. The left hypochondrium contained a 10 cm mass of uncertain origin featuring multiple hypoechoic regions, indicating necrosis (Fig. 1A). The abdominal CT scan indicated multiple uterine fibroids, a left hypochondrium tumor, and retroperitoneal lymphadenopathies in the left external iliac and lateral aortic chains (Fig. 1Ba and-Bb).

**Fig. 2:**
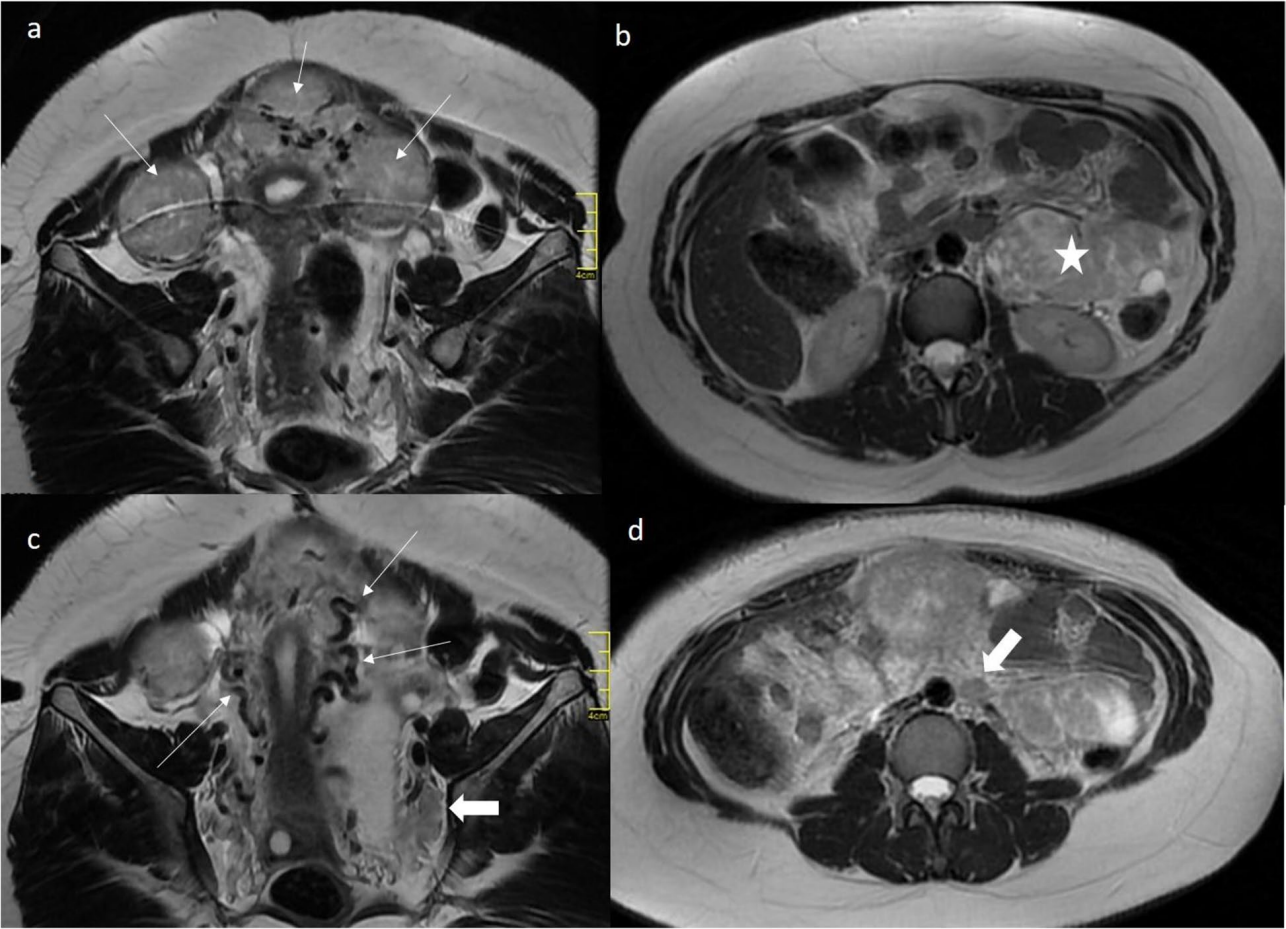
**a** Coronal T2-weighted MRI showing three well-circumscribed hypointense intramural and subserosal leiomyomas (thin arrows), **b** Axial T2-weighted MRI showing interloop and left kidney polypoid mass (indicated by ★) and (**c**) lymphadenopathies in left iliac chain (thick arrows). The bridging vessels sign confirms the uterine origin of the mass (thin arrows), (**d**) lymphadenopathie in lateroaortic chain (thick arrow)

The chest CT showed numerous pulmonary nodules in both lungs (Fig. 1Bc). MRI showed an enlarged asymmetrical myomatous uterus. T2-weighted fast SEMR images showed a normal endometrial stripe and junctional zone with multiple well-circumscribed intramural and subserosal leiomyomas, consistent with type 5 and 6 lesions, according to the International Federation of Gynecology and Obstetrics (FIGO) leiomyoma subclassification system (Fig. 2a). Leiomyomas showed moderate signal intensity compared to the uterine wall with no necrosis or bleeding. MRI also revealed a 10 cm tumor of undefined origin in the left hypochondrium (Fig. 2b) with retroperitoneal lymphadenopathies of the left external iliac and lateral aortic chains (Fig. 2c, d). An axial T1-weighted MR scan showed fibroids with high contrast enhancement and no non-enhancing areas. The bridging vessels sign indicated the uterine origin of masses (Fig. 2c).

Since these imaging results were inconclusive and alarming for metastatic uterine sarcoma or digestive malignancy, we performed a diagnostic and interventional laparotomy in collaboration with the digestive surgery team. The intraoperative findings included: 1) subsural and intramural masses with macroscopic uterine fibroids; 2) normal ovaries and tubes; 3) a polylobed solid mass friable in some areas and adhering only to the visceral peritoneum in the left hypochondrium; and 4) lymphadenopathy in the left external iliac and lateral aortic chain, indicating metastases. As a result, we underwent interannexial hysterectomy, total left hypochondrium mass removal, and lymph node dissection.

**Fig. 3:**
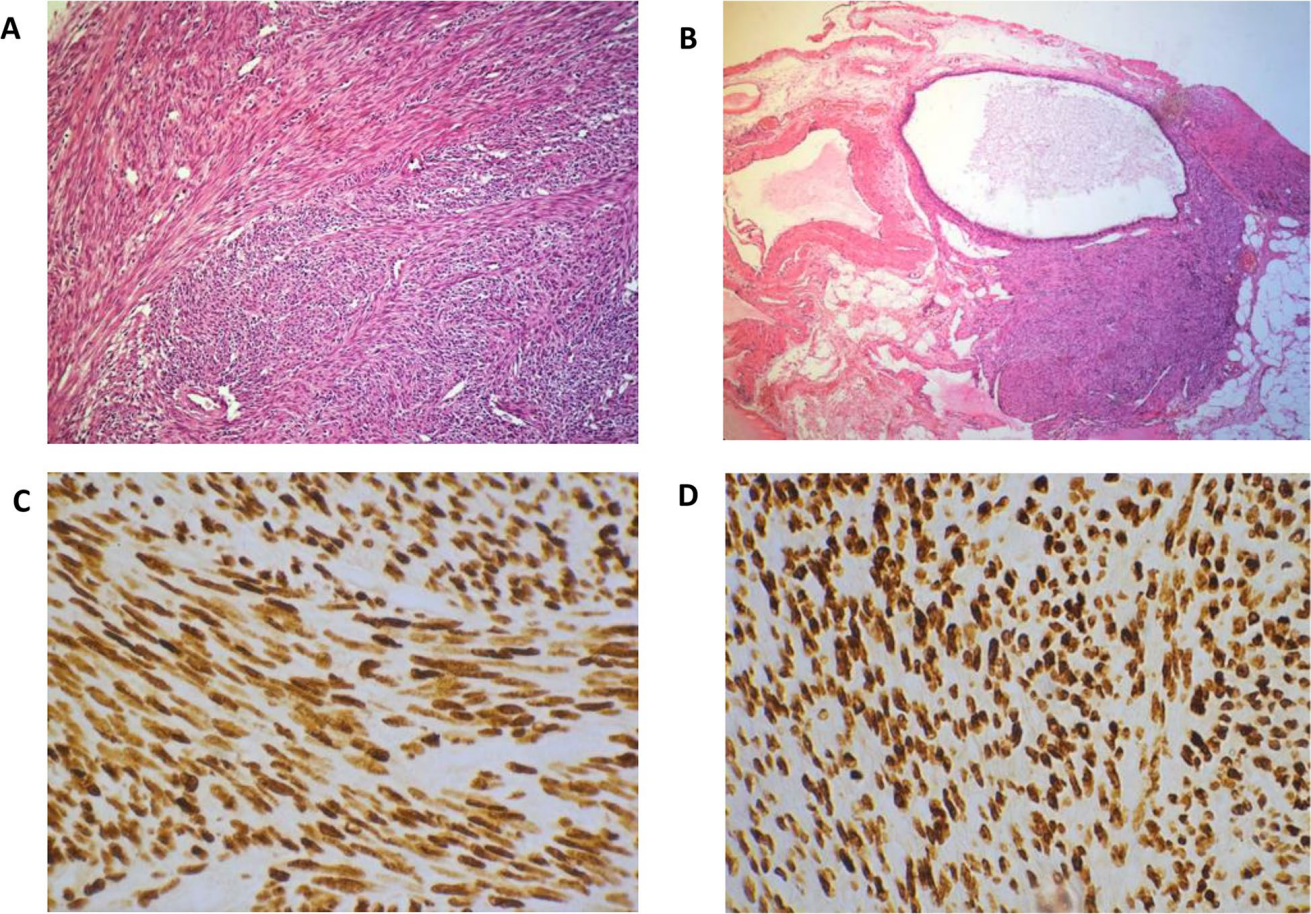
**A** The original uterine leiomyoma demonstrating a typical morphology with bland smooth muscle cells arranged in a fascicular pattern with absence of atypia and mitotic activity (hematoxylin and eosin staining, magnification, × 250). **B** Peritoneal mass showing a coelomic remnant with peripheral muscular differentiation (hematoxylin and eosin staining, magnification × 100). (**C**) Diffuse and strong positivity for estrogen (magnification × 400), (D) progesterone receptors (magnification × 400) throughout the peritoneal tumor

Anatomopathological examination of the uterine masses revealed benign proliferation of smooth muscle cells, suggestive of a leiomyoma (Fig. 3A). The cell nuclei were hyperchromatic and elongated, and lacked signs of atypia or abnormal mitotic processes. The smooth muscle cells had a fascicular organization, separated by a well-vascularized fibro-hyalin stroma without any necrotic zones (Fig. 3A). The cellularity of the left hypochondrium mass resembled that of uterine leiomyoma (Fig. 3B). There was no necrosis or hemorrhage, and it was composed of fascicular smooth muscle cells (Fig. 3B). Lymph nodes removed from the left external iliac and lateral aortic chains revealed massive metastasis of smooth muscular tissue that nearly totally replaced the usual lymphatic tissue with only a few lymph nodes retaining the usual structure. This lymph node smooth muscle tissue resembled uterine leiomyoma and left hypochondriac tumor.

**Fig. 4:**
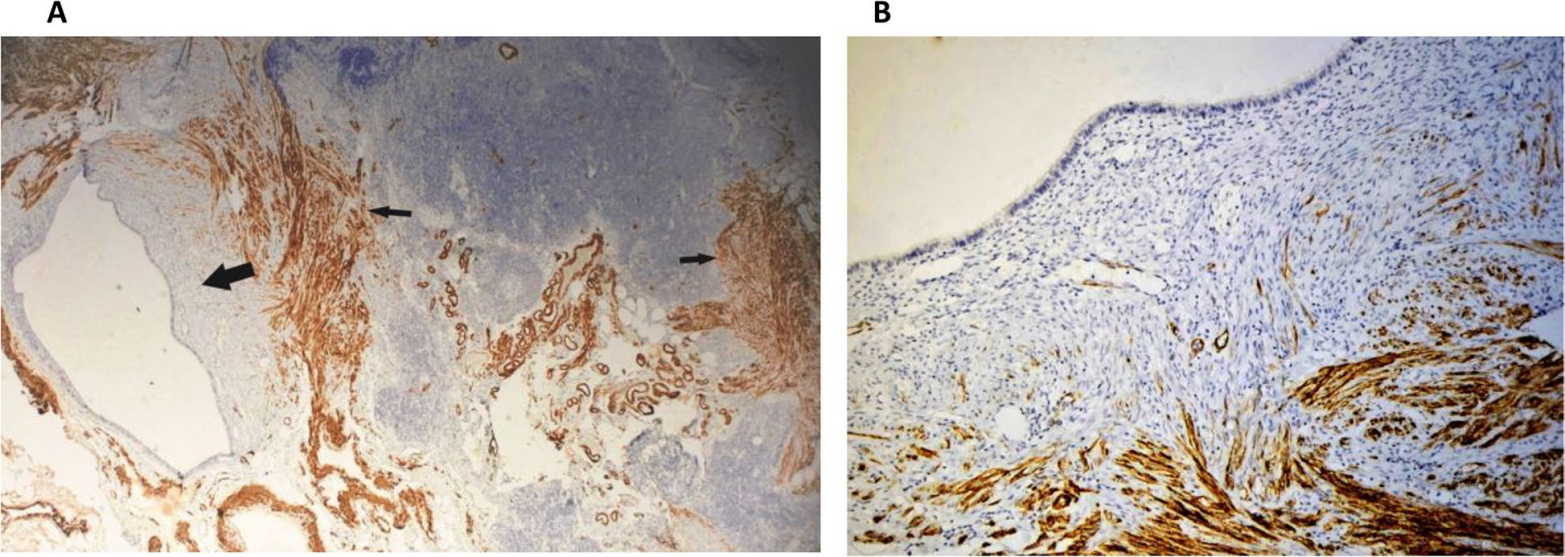
**A** Immunohistochemical staining with an h-caldesmon antibody was performed on the iliac lymph node, which showed strong positivity in differentiated muscular cells (thin arrows). The intranodal coelomic tissue (thick arrow) is negative for the muscular marker (h-caldesmon, × 100). **B** A progressive differentiation of coelomic remnants into muscular tissue (h-caldesmon, magnification × 250)

**Fig. 5:**
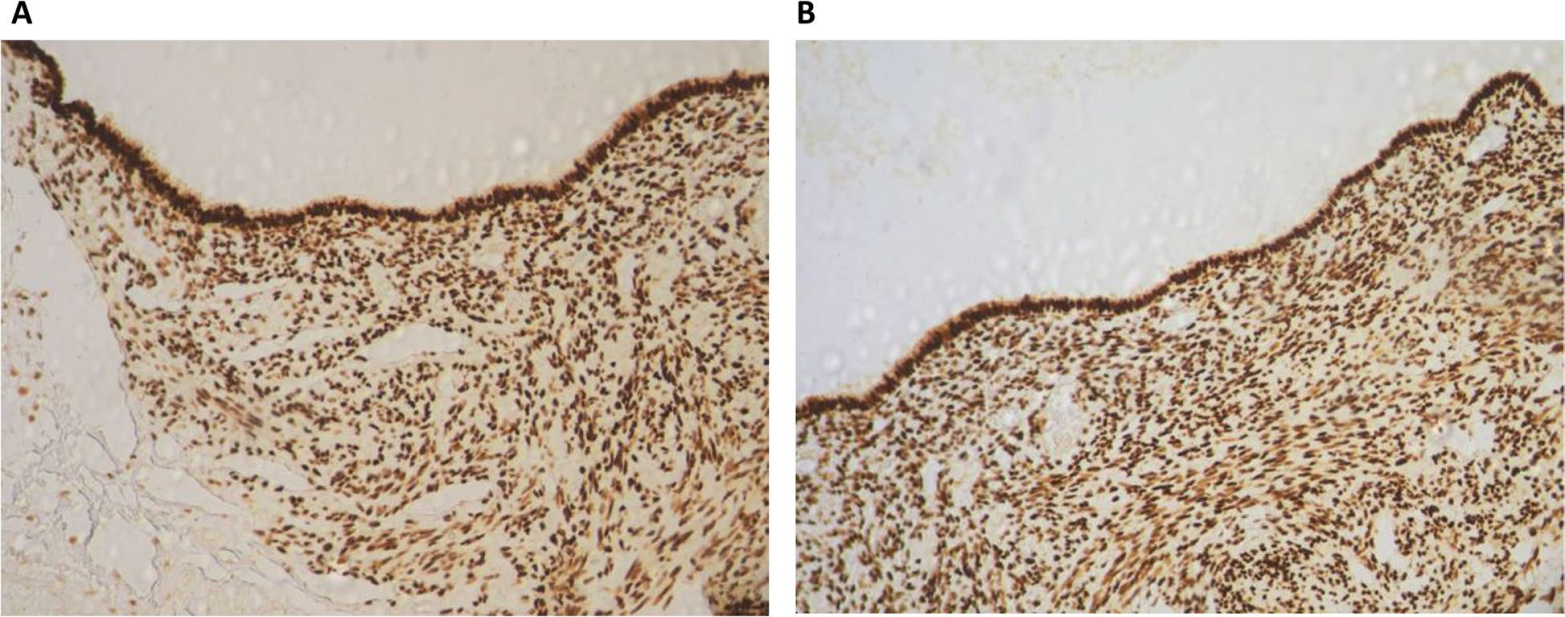
**A** Immunohistochemical staining in an iliac lymph node showing homogeneous strong and diffuse positivity in both the coelomic tissue and muscular foci for estrogen (magnification × 250), **B**, and progesterone receptors (magnification × 250)

The lymph nodes, left hypochondriac tumor, and uterine leiomyoma were immunohistochemically analyzed. All tissues tested were positive for h-caldesmon, smooth muscle actin (SMA), estrogen (ER), and progesterone receptors (PR) (Fig. 3C and D). Importantly, a major finding was made during the immunohistochemical examination of the peritoneal mass (Fig. 3B) and one of the resected lymph nodes (Fig. 4A), which revealed coelomic tissue adjacent to the muscle proliferation within these metastatic sites. This tissue is composed of a columnar coelomic epithelium resting on a moderately cellular mesenchymal tissue (Figs. 3B, 4A and B). The surrounding muscle proliferation was highly positive for smooth muscle actin and h-caldesmon, while the coelomic tissue was negative (Fig. 4A and B). Both the coelomic tissue and muscle foci of the iliac lymph node demonstrated diffuse robust and homogenous estrogen and progesterone receptor positivity (Fig. 5A and B).

Based on the clinical presentation, imaging studies, histology, immunohistochemical positive stains, and Ki-67 index less than 1%, we retained the diagnosis of BML involving the lungs, abdomen, and retroperitoneal lymph nodes and decided to monitor the patient without hormonal therapy.

## Discussion

BML is a rare disorder with an unexplained etiology that affects middle-aged women with a history of uterine leiomyomas. The disease may be caused by histologically benign uterine leiomyoma metastases. Multiple smooth-muscle nodules located in distant extrauterine regions characterize this condition [5–7]. To date, less than 200 cases have been reported in the literature [5, 9–12]. The mean age at the time of diagnosis is 47.3 years [5–7] and the mean interval between the primary surgery (for uterine leiomyoma) and the diagnosis of BML ranges from 8.8 to 14.9 years [5–7, 9]. As previously stated, our patient had surgery 12 years prior to BML diagnosis. BLM has been identified in patients without surgery [5–7], and intervals as brief as 1 month have been described [11]. Total hysterectomy is the most common indicated procedure for BML [5–7, 9], however, several cases, including our case, have had subtotal hysterectomy and conservative myomectomy.

Metastases may occur in various sites with the lungs being the most commonly reported site [9, 12, 13]. The skin, bones, retroperitoneum, lymph nodes, muscles, pleurae, vessels, brain, and spine may also rarely be involved [13, 14]. Approximately 53% of patients exhibit metastases in more than one location [5–7].

Most patients are asymptomatic and metastases are identified inadvertently in routine imaging or medical investigations [5–7, 9, 13]. The rest of the patients have mild to moderate symptoms from lung involvement or metastases in specific anatomical locations. Despite extensive lung involvement, our patient had no respiratory symptoms, and the BML was detected fortuitously on the occasion of left hypochondrium pain. Ultrasonography, CT, and MRI findings are not specific for BML, hence a definitive diagnosis is usually made through histopathological examination of specimens from the uterus and metastatic nodules. The morphology, molecular and immunohistochemical features are characteristic of benign neoplasms with smooth muscle phenotype and a low tumor cell proliferation index Ki-67 [5–7, 15]. The positivity for estrogen and progesterone receptors, as seen in our case, confirms the gynecological tract origin.

The precise etiopathogenesis of BML is unknown. Low-grade uterine leiomyosarcoma spreading to other organs was initially considered [14]. The absence of malignant histological characteristics and indolent clinical course in most published cases refuted this theory and suggests a different mechanism. Additionally, molecular analysis of BML cases showed that extrauterine metastases are clonally related to uterine leiomyomas [14], but the mechanism by which this benign tumor spreads to extra uterine sites is unknown. Hematogenous spread of leiomyoma tissue following surgery is the most common theory [5–7, 12, 14, 16]. The identical histological, immunohistological, and molecular features in the primary tumor and metastatic nodules support this hypothesis, but the long time interval between surgery and BML diagnosis (often exceeding a decade) disproves it. Similarly, the hypothesis that BML may originate from unintentional peritoneal seeding by uterine leiomyoma fragments during hysterectomy or myomectomy does not hold merit because it cannot explain BML onset in patients who have never had uterine surgery [5–7].

Metaplastic transformation of coelmic tissue is a rarely cited alternate hypothesis for BML [5–7, 12]. This hypothesis states that BML can occur at any site with coelomic epithelium [5–7]. Metaplasia and selective hormonal action cause BML neoplasms from subcoelomic mesenchymal cells differentiating into myofibroblasts [5–7]. The metaplasia hypothesis, unlike the hematogenous theory, states that extra uterine nodules or masses are independent neoplasms that develop metaplastically in organs or tissues with ectopic coelomic tissue. Our thorough histopathological and histochemical studies gave for the first time evidence in favor of this theory and made it more compelling. We showed the existence of ectopic coelomic mesenchymal tissue coexisting with leiomyoma tissue in two extra-uterine sites of BML, namely, a lymph node and a “metastatic” peritoneal nodule. The smooth muscle tissue likely originated in the lymph node by metaplastic transformation of the ectopic coelomic tissue rather than spreading from the uterine leiomyoma.

Embryologically, the female genital tract exhibits symmetrical and bilateral coelomic lining thickenings. Müller's ducts, which arise from a longitudinal fold in the posterior wall of the coelom, dominate the female genital tract. Thus, the coelomic mesenchymal tissue gives rise to the smooth muscle of the myometrium as well as possible leiomyomas that may form later.

The process of pluripotent coelomic mesenchymal tissue differentiating into muscle tissue at a site other than its usual site may also explain the presence of disseminated extrauterine nodules of BML, which were previously thought to be metastases from the uterine leiomyoma. As previously established, the Müllerian system's coelomic multipotent cells can differentiate into specialized epithelium or stroma throughout maturity. Toriyama et al. [17] described endometrial foci in diffuse peritoneal leiomyomatosis (DPL). The authors propose that hormone-induced differentiation of coelomic multipotent stem cells into smooth muscle cells, endometrial glands, and stroma is the main cause of endometriosis foci in DPL lesions [17]. In our case, the ubiquitous presence of estrogen and progesterone receptors in coelomic tissue and surrounding muscle foci suggests that hormonal regulation plays a role in differentiation and growth. The current histological observations and the aforementioned literature, which has been proposed without evidence, confirm the metaplastic pathogenic theory.

From a therapeutic standpoint, no guidelines have been established for the treatment of BML. Resection of metastatic nodules is recommended whenever possible [7, 13]. The presence of estrogen and progesterone receptors in primary tumors and disseminated nodules may support hormonal therapy [11]. Bilateral oophorectomy, gonadotropin-releasing hormone agonists, progesterone, selective estrogen receptor modulators, or aromatase inhibitors can reduce estrogen stimulation. These procedures may be recommended for unresectable metastatic lesions [13, 18]. We decided not to use hormonal therapy in our patient despite the presence of ER and PR in the primary tumor and extrauterine nodules. The patient was symptom-free after the abdominal mass was removed; nevertheless, she is still being monitored.

## Conclusion

In this case, we presented a multi-organ BML case. We have found for the first time histology evidence that metaplasia may cause BML. Despite our novel findings, BML's etiopathogenesis may not be explained by a single concept, requiring further research.

## Data Availability

Not applicable.
